# An association study on PSCA rs2294008 polymorphism and gastric cancer: A meta-analysis

**DOI:** 10.3389/fgstr.2022.944525

**Published:** 2022-10-24

**Authors:** HangLong Li, Qiang Zhao, ShuHan Si, DongKai Wu

**Affiliations:** ^1^ Changzhi Medical College, Changzhi City, Shanxi Province, China; ^2^ Heping Hospital Affiliated to Changzhi Medical College, Changzhi City, Shanxi Province, China

**Keywords:** gene, polymorphism, PSCA, rs2294008, meta-analysis

## Abstract

Studies have confirmed that prostate stem cell antigen (PSCA) rs2294008 C>T polymorphism is related to gastric cancer susceptibility, but some studies have reached the opposite conclusion. In this meta-analysis, we attempted to clear up these differences and explore the relationship between the different factors that influence susceptibility to gastric cancer. Studies with publication dates that preceded 16 April 2022 were selected from PubMed, Springer, EMBASE, and Web of Science, and the relationship between risk models and gastric cancer was analyzed by odds ratios (ORs) and 95% confidence intervals. Consequently, it was confirmed that PSCA rs2294008 polymorphism leads to an increased risk of gastric cancer. Subgroup analysis found that individuals with diffuse gastric cancer, non-cardia gastric cancer, *Helicobacter pylori* (HP)-positive or who are from the white or Asian population faced an increased susceptibility to gastric cancer. Those from the white populations faced significantly higher risks than Asians, and the association of PSCA with gastric cancer could be significantly increased by genome-wide association analysis. However, the conclusion that smoking reduces susceptibility to gastric cancer appears to be abnormal. Further prospective investigations that involve smoking and have a larger sample size are required.

## Introduction

Gastric cancer (GC) has been widely studied for decades due to its extremely high morbidity and mortality rates. According to statistics, in 2020, around 1 million individuals have been diagnosed with gastric cancer and approximately 769,000 people have died of this disease, accounting for 7% of global deaths (1). The diversity of gastric cancer genes is a pivotal factor that hinders the precise treatment of GC. Among them, single nucleotide polymorphism (SNP) is an important cause of gene diversity, and several studies show that SNPs can be used as predictive biomarkers of human genetic variation to detect individual cancer risk.

Prostate stem cell antigen (PSCA) is a protein derived from normal epithelial tissues of the bladder, kidney, esophagus, and stomach (2). SNPs in PSCA are associated with various tumors such as rs1045531 that increases the risk of prostate cancer in Chinese men (2), rs2294008 that increases susceptibility to gastric and bladder cancers (3), and while rs2294008 alters the progesterone receptor status, together with rs2976392, it increases the risk of breast cancer (4). Differences in SNPs can affect the biological behavior of tumors. Therefore, the study of SNPs will help to elucidate the function of PSCA, the low expression of which has been confirmed to be significantly associated with poor overall survival rates in gastric cancer (5). Among the SNPs in PSCA, rs2294008 and rs2976392 have been shown to significantly increase susceptibility to gastric cancer (6). Given that rs2976392 only increases cancer risk in Asian populations, rs2294008 may be more reasonable as a potential universal gastric cancer biomarker.

Several recent meta-analyses of PSCA rs2294008 C>T and GC susceptibility have confirmed the association. However, some of the most recent studies have suggested some different points. Sung et al. (7) found that PSCA has a carcinogenic effect on gastric cancer, but Alikhani et al. (8) never found a correlation between PSCA rs2294008 and gastric cancer. In addition, SNPs are closely related to gender, ethnicity, habits, etc. It has been reported that the prevalence of GC in men was twice that in women in the last century, but this ratio is now being inverted (9). Cai et al. (10) found a synergy between *Helicobacter pylori* (HP) and PSCA, but this information had not been analyzed before. Thus, we conducted this meta-analysis and compared how gender, HP infection, smoker status, and the genotyping methods discussed contribute to or indicate susceptibility to gastric cancer to improve the study of the correlation between PSCA rs2294008 C>T and gastric cancer.

## Materials and methods

### Search strategy

Studies on PSCA rs2294008 C>T polymorphisms were found using PubMed, Springer, EMBASE, and Web of Science and examined to determine the association with gastric cancer risk. Literature on the subject published before 16 April 2022 was included. The search was completed using these keywords: “PSCA” or “prostate stem cell antigen”, “polymorphism” or “SNP” or “gene” and “gastric cancer” or “stomach cancer” or “GC”. Eligible literature was retrieved from all of the publications. Furthermore, we hand-collected additional literature from the reference lists of review articles examined to ensure all potential eligible publications are included.

### Inclusion criteria

The studies were included according to these rules: (1) the research mentions the correlation between PSCA rs2294008 C>T polymorphism and the risk of GC. (2) It meets the requirements of a case-control study. (3) The study provides odds ratios (ORs) and 95% confidence intervals (CIs).; (4) Published literature provides detailed genotype distribution data. (5) The study subjects were gastric cancer patients and healthy people (precancerous lesions and other types of diseases were excluded).

### Data extraction and summary

Data were screened by two fellows, and if any disagreement was unresolved, it was referred to a third researcher. For the record, the following information was extracted from all analyzed data: first author, year, country, ethnicity, control origin (population or hospital), genotyping method [e.g., Taqman, PCR-restriction fragment length polymorphism (RFLP), and Genome-Wide Association Studies (GWAS)], case and control sample quantities, distribution frequency of each genotype, Lauren classification (intestinal and diffuse), anatomical site (cardia and non-cardia), gender, HP infection, smoker status, and Hardy–Weinberg equilibrium (HWE) test results.

### System analysis

Genetic models were formed according to genotype, and the ORs and 95% CIs of each model were determined to evaluate the relationship between PSCA rs2294008 C>T polymorphism and gastric cancer risk. They were then divided according to genotype distribution: the dominant model (CT+TT vs. CC), the recessive model (TT vs. CT+CC), homozygous (TT vs. CC), heterozygous (CT vs. CC), and the allelic (T vs. C). The goodness-of-fit chi-square test was used to test the deviation of the control group from the HWE, with a significant imbalance at the 0.05 level. A chi-square-based Q test was used to estimate heterogeneity in the different studies and the I2 statistic to quantify it. In cases where *P*<0.05 indicates significant heterogeneity between studies, the random-effects model was conducted. In other cases, a fixed-effect model was employed. The sensitivity analysis excluded each study once, and the summary ORs with 95% CIs were recalculated to determine how stable the summary data were. Begg’s and Egger’s tests were used to determine publication bias. Every statistic was performed using the Stata 15.0 software.

## Results

### Research characteristics

**Figure 1:**
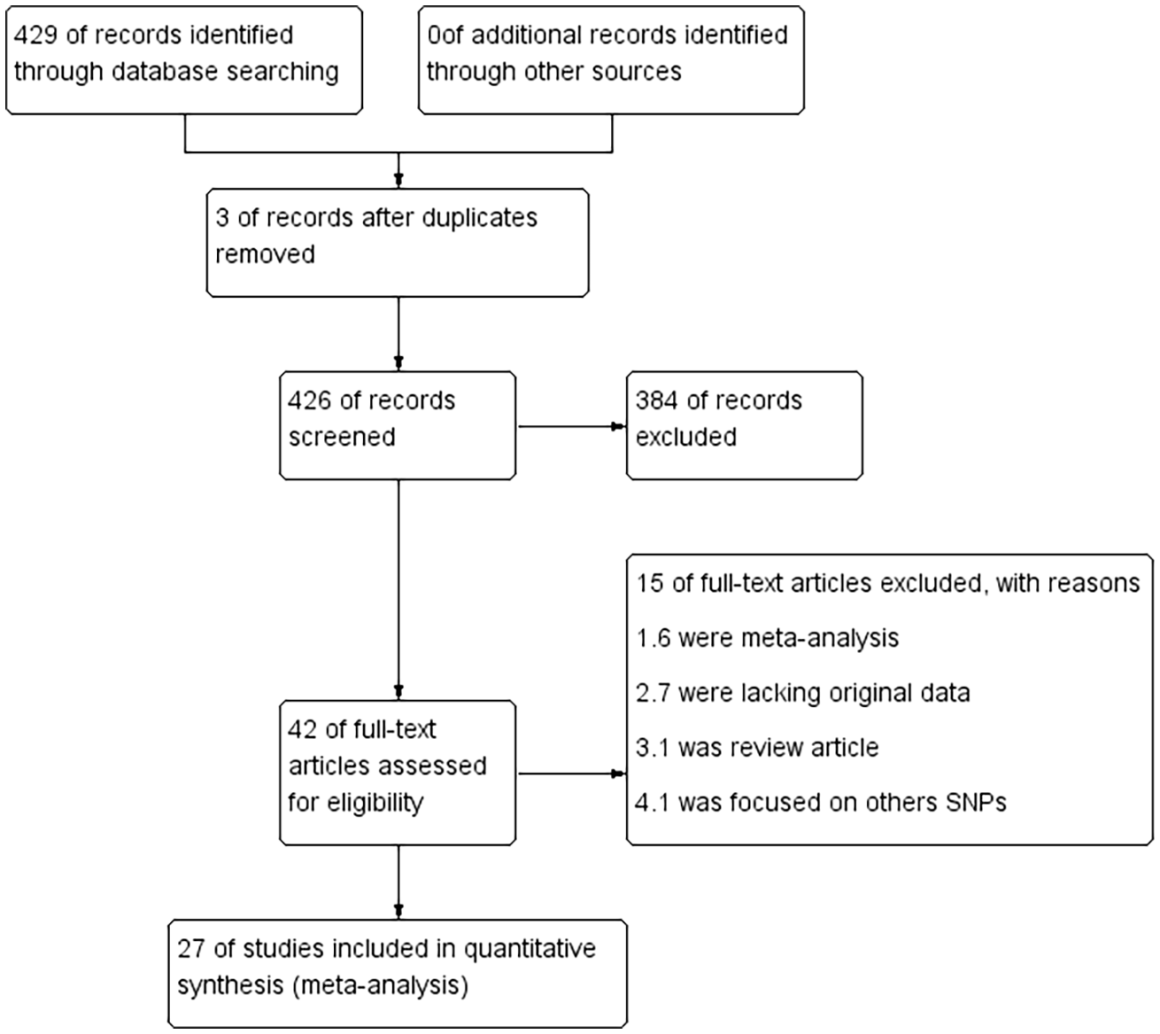
Literature search and screening flowchart.

This meta-analysis included 28 eligible literature articles and 18,495 patients and 19,219 healthy controls (7, 8, 10–33). Specific information on the included literature is shown in **Table 1**. Except for five articles, the control genotypes of all related studies had *P*-values higher than 0.05 in Hardy–Weinberg equilibrium (HWE), which is consistent with the genetic equilibrium in Mendelian populations. **Figure 1** shows the process of searching for and selecting literature.

**Table 1:** Study characteristics included in the meta-analysis.

Reference							Case			Control			
	Country	Ethnicity	SOC	Genotyping	Case	Control	CC	CT	TT	CC	CT	TT	HWE
Lochhead et al. (2011) (11)	USA	White	PB	Taqman	308	208	85	129	94	49	110	49	0.405
Lochhead et al. (2011) (11)	Poland	White	PB	Taqman	292	382	47	143	102	101	166	115	<0.05
Sala et al. (2012) (12)	Europe	White	PB	Taqman	409	1,515	93	198	118	491	714	310	0.0884
Rizzato et al. (2013) (13)	Germany	White	PB	Taqman	178	1,057	23	86	69	231	507	319	0.2692
Kupcinskas et al. (2014) (14)	Lithuania	White	HB	Taqman	251	243	33	116	102	64	123	56	0.834
Sun et al. (2014) (15)	USA	White	HB	Taqman	130	125	17	64	49	30	63	32	0.9264
Garcia-Gonzalez et al. (2015) (34)	Spain	White	HB	Taqman	603	675	154	302	147	199	346	130	0.3495
Gonzales-Hormazabal et al. (2020) (16)	Chile	Other	HB	Other	307	310	110	162	35	87	156	67	0.851
The Study Group of Millennium Genome Project for Cancer et al. (2008) (17)	Korea	Asian	HB	Taqman	871	390	133	461	277	122	176	92	0.069
The Study Group of Millennium Genome Project for Canceret al. (2008) (17)	Japan	Asian	HB	GWAS	1,524	1,396	96	700	728	210	650	536	0.5738
Wu et al. (2009) (18)	China	Asian	PB	PCR-RFLP	1,710	995	759	819	132	506	412	77	0.5865
Matsuo et al. (2009) (19)	Japan	Asian	HB	Taqman	708	708	330	329	49	273	338	97	0.6378
Ou et al. (2010) (20)	China	Asian	HB	PCR-RFLP	196	246	85	93	18	132	96	18	0.9243
Lu et al. (2010) (21)	China	Asian	PB	PCR-RFLP	1,023	1,069	547	404	72	605	387	77	0.1664
Zeng et al. (2011) (22)	China	Asian	HB	PCR-RFLP	460	549	202	216	42	289	223	37	0.4932
Song et al. (2011) (23)	Korea	Asian	HB	PCR-RFLP	3,239	1,700	570	1620	1049	414	818	468	0.1305
Li et al. (2012) (24)	China	Asian	PB	Other	300	300	124	141	35	168	111	21	0.6501
Zhao et al. (2013) (25)	China	Asian	PB	Other	717	951	275	342	100	465	401	85	0.9127
Ichikawa et al. (2015) (26)	Japan	Asian	HB	PCR-RFLP	193	266	24	104	65	52	119	95	0.1851
Zhang et al. (2015) (27)	China	Asian	HB	Other	475	480	227	207	41	261	183	36	0.6177
Mou et al. (2015) (28)	China	Asian	PB	Other	198	130	23	126	49	5	34	91	0.4257
Turdikulova et al. (2016) (29)	Uzbekistan	Asian	HB	PCR-RFLP	268	248	78	190	0	119	109	20	0.4721
Qiu et al. (2016) (30)	China	Asian	HB	Taqman	1,124	1,192	537	489	98	663	383	146	<0.05
Sun et al. (2015) (31)	China	Asian	HB	Taqman	702	774	332	309	61	405	297	72	0.1052
Cai et al. (2017) (10)	China	Asian	PB	PCR-RFLP	485	488	215	225	45	268	173	47	<0.05
Sung et al. (2017) (7)	China	Asian	HB	GWAS	194	170	24	112	58	54	84	32	0.9473
Yan et al. (2019) (32)	China	Asian	HB	Taqman	549	592	269	236	44	337	191	64	<0.05
Alikhani et al. (2020) (8)	Iran	Asian	HB	PCR-RFLP	99	96	62	29	8	63	24	9	<0.05
Guan (2020) (33)	China	HB	Asian	Other	982	1,964	615	337	30	1,071	744	149	0.2115

SOC, source of control; HB, hospital-based controls; PB, population-based controls; GWAS, Genome-Wide Association Studies; PCR-RFLP, Polymerase Chain Reaction-restriction fragment length polymorphism; HWE, Hardy-Weinberg equilibrium.

Among these studies, there are seven on white populations, 21 on Asians, and one on the Latin-American population. These populations were studied using the Taqman, Polymerase Chain Reaction-restriction fragment length polymorphism, Genome-Wide Association Studies, MassARRAY, and six other methods for genotyping. To differentiate control sources, they were further grouped into population-based (population-based controls) and hospital-based (hospital-based controls), totaling 10 PBs and 19 HBs. In addition, we used Lauren classification, anatomical location, gender, HP infection, and smoker status as subgroups to study their effects on PSCA rs2294008 C>T polymorphism, including 12 anatomical subgroups with clear genotyping—six per gender and smoker status, and then four per Lauren classification and HP infection subgroup—were used.

### Quantitative synthesis results

**Figure 2:**
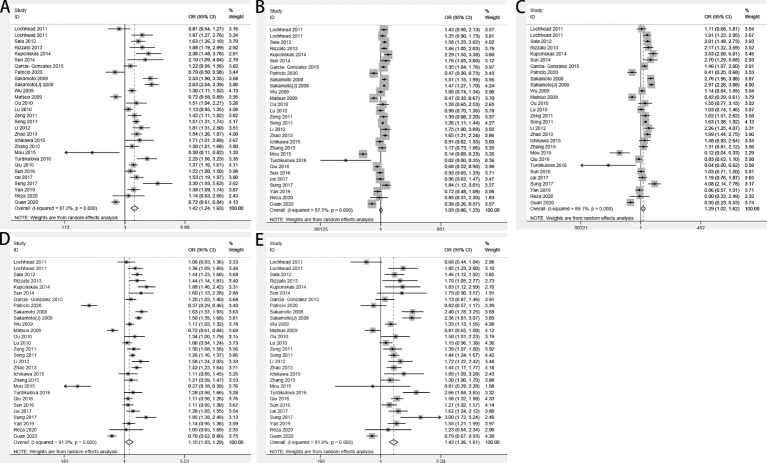
Forest plots of the relationship between prostate stem cell antigen (PSCA) rs2294008 C>T and GC risk. **(A)** CT+TT vs. CC; **(B)** TT vs. CT+CC; **(C)** TT vs. CC; **(D)** T vs. C; and **(E)** CT vs. CC.

By pooling all of the data together (**Table 2**), the polymorphism of PSCA rs2294008 C>T was associated with an increased risk of GC (CT+TT vs. CC: OR=1.42, 95% CI: 1.24–1.63; TT vs. CT +CC: OR=1.03, 95% CI: 0.86–1.23; TT vs. CC: OR=1.29, 95% CI: 1.02–1.62; CT vs. CC: OR=1.43, 95% CI: 1.26–1.61; and T vs. C: OR=1.15, 95% CI: 1.03–1.29) (**Figure 2**). Stratified by ethnicity, PSCA rs2294008 was most significant in white populations (CT+TT vs. CC: OR=1.56, 95% CI: 1.01–1.20; TT vs. CT+CC: OR=1.51, 95% CI: 1.32–1.73; TT vs. CC: OR=1.92, 95% CI: 1.49–2.48; CT vs. CC: OR=1.38, 95% CI: 1.07–1.78; and T vs. C: OR=1.38, 95% CI: 1.22–1.57). From the subgroup analysis of genotyping technology, Genome-Wide Association Studies was very significant in the detection of related cancer susceptibility (CT+TT vs. CC: OR=2.74, 95% CI: 2.18–3.45; TT vs. CT+CC: OR=1.49, 95% CI: 1.30–1.72; TT vs. CC: OR=3.11, 95% CI: 2.43–3.98; CT vs. CC: OR=2.46, 95% CI: 1.94–3.13; and T vs. C: OR=1.59, 95% CI: 1.33–1.91). Furthermore, we found that the PSCA rs2294008 C>T polymorphism was associated with GC anatomical location, Lauren classification, HP infection status, and smoker status, while gender was not significantly associated with PSCA rs2294008 expression.

**Table 2:** Meta-analysis of prostate stem cell antigen (PSCA) rs2294008 association with gastric cancer subgroups.

Variables	No. of studies	Homozygous (TT vs. CC)			Heterozygous(CT vs. CC)			Recessive [TT vs. (CT+CC)]			Dominant [(CT+TT) vs. CC]			Allelic (Comparing T vs. C)		
		OR(95% CI)	P eff	P het	OR (95% CI)	P eff	P het	OR (95% CI)	P eff	P het	OR (95% CI)	P eff	P het	OR (95% CI)	P eff	P het
*All*	28	1.36 (1.09, 1.69)	0.006	<0.00001	1.46 (1.31, 1.63)	<0.001	<0.00001	1.07 (0.90, 1.27)	0.428	<0.00001	1.46 (1.29, 1.65)	<0.001	<0.00001	1.17 (1.05, 1.31)	0.004	<0.00001
*Ethnicity*																
White	7	1.92 (1.49, 2.48)	<0.001	0.033	1.38 (1.07, 1.78)	0.013	0.009	1.51 (1.32, 1.73)	<0.001	0.333	1.56 (1.21, 2.01)	0.001	0.004	1.38 (1.22, 1.57)	<0.001	0.029
Asian	20	1.26 (0.97, 1.64)	0.087	<0.00001	1.52 (1.35, 1.73)	<0.001	<0.00001	0.97 (0.78, 1.20)	0.76	<0.00001	1.48 (1.28, 1.70)	<0.001	<0.00001	1.18 (1.05, 1.31)	0.004	<0.00001
Other	1	0.41 (0.25, 0.68)	<0.001	/	0.82 (0.57, 1.17)	0.28	/	0.47 (0.30, 0.73)	/	/	0.70 (0.50, 0.98)	/	/	0.37 (0.29, 0.46)	<0.001	/
*Genotyping*																
Taqman	12	1.45 (1.03, 2.05)	0.035	<0.00001	1.40 (1.15, 1.70)	0.001	<0.00001	1.17 (0.92, 1.50)	0.203	<0.00001	1.45 (1.17, 1.79)	0.001	<0.00001	1.26 (1.09, 1.45)	0.002	<0.00001
PCR-RFLP	9	1.30 (1.05, 1.60)	0.016	0.214	1.47 (1.28, 1.69)	<0.001	0.015	1.07 (0.89, 1.28)	0.475	0.06	1.43 (1.27, 1.61)	<0.001	0.044	1.21 (1.15, 1.28)	<0.001	0.618
GWAS	2	3.11 (2.43, 3.98)	<0.001	0.374	2.46 (1.94, 3.13)	<0.001	0.443	1.49 (1.30, 1.72)	<0.0001	0.389	2.74 (2.18, 3.45)	<0.001	0.457	1.59 (1.33, 1.91)	<0.001	0.195
Other	5	0.84 (0.37, 1.93)	0.685	<0.00001	1.26 (0.99, 1.62)	0.064	0.03	0.74 (0.30, 1.83)	0.516	<0.00001	1.11 (0.76, 1.63)	0.582	<0.00001	0.78 (0.42, 1.44)	0.421	<0.00001
*SOC*																
PB	10	1.36 (1.01, 1.84)	0.042	<0.00001	1.37 (1.19, 1.58)	<0.001	0.012	1.06 (0.75, 1.50)	0.737	<0.00001	1.39 (1.18, 1.64)	<0.001	<0.00001	1.15 (0.96, 1.38)	0.118	<0.00001
HB	18	1.37 (1.01, 1.85)	0.042	<0.00001	1.52 (1.30, 1.78)	<0.001	<0.00001	1.08 (0.88, 1.31)	0.469	<0.00001	1.52 (1.27, 1.81)	<0.001	<0.00001	1.19 (1.03, 1.37)	0.019	<0.00001
*Subgroup by location*																
Cardia	6	1.13 (0.75, 1.70)	0.572	0.135	0.95 (0.65, 1.39)	0.778	0.039	1.17 (0.90, 1.51)	0.232	0.496	0.99 (0.68, 1.44)	0.969	0.031	1.05 (0.85, 1.30)	0.629	0.095
Non-cardia	6	1.71 (1.38, 2.12)	<0.0001	0.493	1.27 (1.03, 1.56)	0.023	0.228	1.53 (1.28, 1.83)	<0.0001	0.501	1.40 (1.18, 1.65)	<0.0001	0.36	1.33 (1.19, 1.48)	<0.0001	0.362
*Subgroup by histology*																
Intestinal	12	1.39 (1.01, 1.91)	0.046	<0.00001	1.34 (1.11, 1.62)	0.002	<0.00001	1.17 (0.95, 1.44)	0.13	<0.00001	1.37 (1.10, 1.70)	0.004	<0.00001	1.21 (1.05, 1.41)	0.01	<0.00001
Diffuse	12	2.44 (1.63, 3.65	<0.0001	<0.00001	1.71 (1.26, 2.33)	0.001	<0.00001	1.67 (1.35, 2.06)	<0.0001	0.001	1.91 (1.38, 2.66)	<0.0001	<0.00001	1.52 (1.29, 1.79)	<0.0001	<0.00001
*Helicobacter pylori*																
Positive	3	1.20 (0.83, 3.14)	0.966	<0.00001	1.17 (0.80, 1.71)	0.426	0.007	1.26 (0.71, 2.35)	0.84	<0.00001	1.20 (0.70, 2.06)	0.507	<0.00001	1.10 (0.69, 1.76)	0.689	<0.00001
Negative	3	1.08 (0.39, 2.96)	0.884	<0.00001	1.14 (0.66, 1.98)	0.762	0.041	1.08 (0.51, 2.30)	0.839	0.004	1.01 (0.55, 1.82)	0.99	0.002	1.03 (0.62, 1.72)	0.913	<0.00001
*Sex*																
Male	6	1.02 (0.37, 2.85)	0.97	<0.00001	1.23 (1.03, 1.46)	0.021	0.060	0.96 (0.44, 2.12)	0.925	0.001	/	/	/	1.02 (0.65, 1.61)	0.915	<0.00001
Female	6	0.58 (0.18, 1.84)	0.353	0.015	1.19 (0.95, 1.49)	0.127	0.220	0.59 (0.18, 1.95)	0.391	0.007	1.13 (0.87, 1.47)	0.344	0.078	0.88 (0.56, 1.38)	0.587	0.047
*Smoker*																
Never	6	1.37 (0.36, 5.25)	0.647	<0.00001	1.35 (1.08, 1.69)	0.009	0.033	1.01 (0.41, 2.49)	0.991	0.001	1.50 (0.67, 3.38)	0.323	<0.00001	1.93 (1.26, 2.95)	0.002	0.020
Ever	6	1.21 (0.41, 3.58)	0.737	<0.00001	1.16 (0.97, 1.38)	0.102	0.169	1.16 (0.50, 2.66)	0.734	<0.00001	1.18 (0.93, 1.50)	0.161	0.012	1.14 (0.69, 1.88)	0.621	<0.00001

No., number; vs., versus; OR, odds ratio; CI, confidential interval; PEff, p-value of pooled effect; PHet, p-value of heterogeneity test.

### Sensitivity and heterogeneity analyses

The analysis of the included studies revealed significant heterogeneity (CT+TT vs. CC: *P*<0.00001, I ^2 =^ 87.5%; TT vs. CC+CT: *P*<0.00001, I ^2 =^ 87.5%; TT vs. CC: *P*<0.00001, I ^2 =^ 89.1%; CT vs. CC: *P*<0.00001, I ^2 =^ 81.9%; and T vs. C: *P*<0.00001, I ^2 =^ 91.9%). Sensitivity analyses were repeated, ignoring one study per repetition in order to identify sources of heterogeneity. The results showed that the main sources of heterogeneity were Mou et al. (28), Turdikulova et al. (29), and Gonzalez-Hormazabal et al. (16). The heterogeneity was significantly reduced after excluding the above three studies (CT+TT vs. CC: *P*<0.00001, I ^2 =^ 80.5%; TT vs. CC+CT: *P*<0.00001, I ^2 =^ 74.9%; TT vs. CC: *P*<0.00001, I ^2 =^ 83.0%; CT vs. CC: *P*<0.00001, I ^2 =^ 72.9%; and T vs. C: *P*<0.00001, I ^2 =^ 74.0%).

### Publication bias

**Figure 3:**
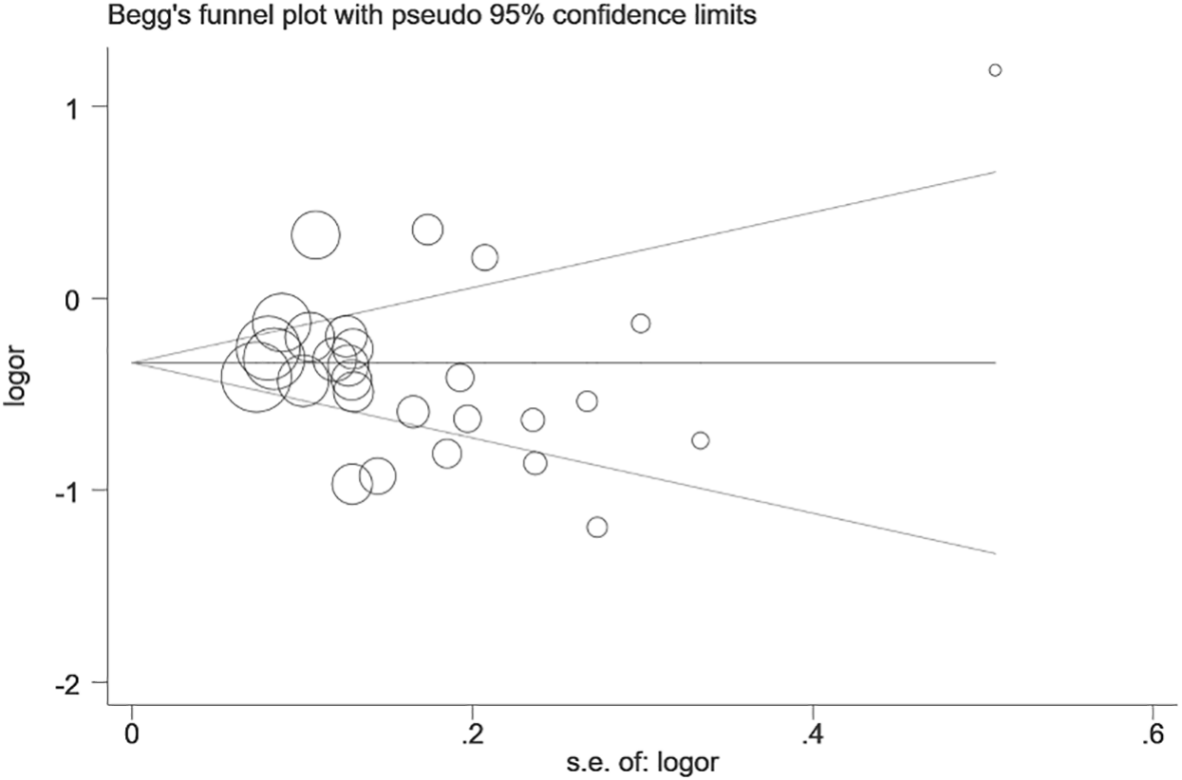
Begg’s funnel plot of publication bias test of prostate stem cell antigen (PSCA) rs2294008 C>T polymorphism (CT+TT vs. CC).

We performed statistical analysis on publication bias in the included literature. The result is shown in **Figure 3**. Begg’s funnel plot for the dominant model is symmetrical, and no significant bias is shown, which was confirmed by Egger’s test (dominant: *P*=0.180, recessive: *P*=0.106, homozygous: *P*=0.272, heterozygous: *P*=0.141, and allelic: *P*=0.640).

## Discussion

Prostate stem cell antigen (PSCA) is a protein that binds to the cell surface, and the two are linked by glycosylphosphatidylinositol (GPI). PSCA is found in the human bladder, prostate epithelial cells (35), and stomach, and for the latter, is expressed in the isthmus of the gastric gland. Studies have shown that there may be proliferative precursor cells in the isthmus, and PSCA can act on those cells to negatively affect tumor proliferation (17). Xu et al. (35) detected by Western blotting that the content of PSCA in GC was much lower than that in normal gastric mucosa. With *in vitro* functional experiments, silencing PSCA could promote the proliferation of GC cells, and overexpression would lead to inhibition. In the previous meta-analysis (6), it was confirmed that the PSCA rs2294008 C>T polymorphism level was significantly raised in GC.

In this updated meta-analysis, with a total of 28 studies containing 18,495 patients and 19,219 healthy controls, we confirmed that PSCA rs2294008 was clearly associated with GC susceptibility, particularly evident in dominant models. Sakamoto et al. (17) first found that rs2294008 in Asian populations regulates the transcriptional activity upstream of PSCA and has been shown to act on the proliferation process of gastric cancer. Subsequently, Sala et al. (12) also found that PSCA rs2294008 SNP in white populations has the same risk genotype. Our meta-analysis found that in white and Asian populations TT genotypes are strongly associated with the former whereas TC genotypes are associated with cancer risk in Asians. The difference in association between the ethnicities indicates that genetic information can significantly affect SNPs. Whether different genotypes are the main factors affecting the differences in the susceptibility to gastric cancer among ethnic groups needs to be evaluated further. Gonzalez-Hormazabal et al. (16) found for the first time that PSCA rs2294008 also increased the risk of cancer in Chilean populations, which indicated that PSCA rs2294008 could be seen as a biomarker to estimate the genetic risk of gastric cancer beyond Asian and white populations.

As genotyping techniques continue to upgrade, whether the accuracy of various techniques causes bias is also an issue to be discussed, and the previous five meta-analyses have not analyzed them. Therefore, we have collected six techniques, namely, Taqman, PCR-RFLP, Genome-Wide Association Studies, MassARRAY, DHPLC, and Sequenom, among which 12 studies used Taqman, nine studies used PCR-RFLP, and two studies used Genome-Wide Association Studies. Classified as “Other,” this meta-analysis found statistical significance for all five genotype models. In addition, the correlation between PSCA rs2294008 C>T and GC has been confirmed *via* Taqman, PCR-RFLP, and Genome-Wide Association Studies, among which GWAS showed a very significant correlation. However, in-depth studies have found that a single SNP usually shows only moderately on the GWAS platform effect. A single SNP usually only has marginal effects, ignoring epistatic effects between SNPs, which can significantly contribute to disease susceptibility (18). Taqman has the characteristics of high precision and low cost in the study of single SNP and large sample populations (36). Therefore, we recommend genotyping with GWAS when performing susceptibility analysis on SNP pools and Taqman genotyping on individual SNPs.

Since PSCA is expressed in the gastric gland isthmus, anatomical factors may influence the location of GC. In this meta-analysis, the effect of PSCA rs2294008 on non-cardia gastric cancer was significantly higher than that of cardia gastric cancer, which was consistent with the results of previous studies (32, 37). Similarly, the Lauren classification analysis showed that the disease susceptibility of the diffuse type was higher than that of the intestinal type. In addition, it was found that gender was not correlated with PSCA rs2294008 in the gender analysis, which is opposite to the results of Qiu et al. (30). Considering that the included studies are basically from Asia, a larger gender subgroup analysis is required to validate this finding. Smoking is a well-established risk factor of GC, compared with non-drinkers and non-smokers, alcoholics and smokers have a two-fold danger of gastric cancer (38). However, the study found that in all models, non-smokers are more susceptible than smokers, and this difference needs careful analysis.

Previous researchers have reported that a group with *H. pylori* infection in the same PSCA model may have a greater GC susceptibility than HP-negative people (10), Tanikawa et al. (39) found that the growth-promoting effect of the PSCA rs2294008 T gene may increase the risk of HP-induced GC, possibly because the PSCA protein encoded by the T gene has a 9-amino acid fragment attached to the N-terminus, which is longer than the synthetic protein of the C gene, increasing the risk of cancer by facilitating epithelial cell proliferation (40). A recent study showed that HP infection had a stronger effect on PSCA rs2294008 T carriers, whose PSCA expression was significantly suppressed compared with HP-negative normal (41). These studies support our study that the T gene susceptibility was higher in HP-positive gastric cancer patients than that of the C gene, and the susceptibility of HP-positive patients is higher than that of HP-negative patients in all models. We speculate that HP interacts with PSCA to increase the risk of cancer. However, there are too many susceptibility factors for HP, and the effect of PSCA on such factors needs further study.

We found in the sensitivity analysis that the heterogeneity was significantly reduced after removing the three studies by Mou et al., Turdikulova et al., and Gonzalez-Hormazabal et al. By contrast, Mou et al. confirmed that PSCA rs2294008 was not associated with GC risk, contrary to most results. The study by Turdikulova et al., conducted primarily in Uzbekistan, found that only the CT genotype was associated with significant cancer risk, whereas Asian risk models were susceptible to both TT and CT genotypes. The study by Gonzalez-Hormazabal et al. showed that gastric cancer patients in Chile were not statistically significant in the stealth model, and in a study focusing on Brazil, PSCA rs2976392 was significantly associated with GC risk, we speculate that gastric cancer susceptibility in Latin-American populations may be related to rs2976392, not rs2976392 and rs2294008, which also needs to be confirmed by controlled studies in this population.

In this meta-analysis, we analyzed the association between PSCA rs2294008 and gastric cancer and also analyzed the factors that may influence the association, such as ethnicity, Lauren classification, anatomical site, and gender, and the results were consistent with previous meta-analyses (6, 42). However, the previous analysis did not evaluate the susceptibility factors of gastric cancer, making the evidence of rs2294008 as biomarkers insufficient, while HP infection and smoker status remain key factors in predicting gastric cancer. Therefore, we performed the first statistical analysis of the role of both factors in rs2294008 and gastric cancer, and the results were encouraging. HP infection and rs2294008 correlation was higher than without HP infection, and this new finding may complement the influence of HP in gastric cancer biology and further improve the evidence for rs2294008 as a gastric cancer biomarker.

## Conclusion

In conclusion, PSCA rs2294008 C>T can significantly increase susceptibility to GC, especially in Asian and white populations, and non-cardia gastric cancer, diffuse gastric cancer, and HP infection. However, the result that smoking does not increase susceptibility to gastric cancer seems abnormal. Due to the limited number of samples, further prospective studies involving smoking with a more extensive sample size are needed to clarify.

## Data availability statement

The original contributions presented in the study are included in the article/**Supplementary Material**. Further inquiries can be directed to the corresponding author.

## Ethics statement

Ethical review and approval were not required for this study according to local legislation and institutional requirements. The participants provided their written informed consent to participate in this study. Written informed consent was obtained from the relevant individual(s) for the publication of any potentially identifiable images or data included in this article.

## Author contributions

HL performed the data analysis and literature writing; the SS searched all relevant literature; DW contributed to the data analysis; and QZ contributed to the research process. All authors contributed to the article and approved the submitted version.

## Funding

This work was supported by a grant from the Changzhi Medical College Academic Leader Program. (KS202003)

## Acknowledgments

We thank Changzhi Medical College for supporting this research.

## Conflict of interest

The authors declare that the research was conducted in the absence of any commercial or financial relationships that could be construed as a potential conflict of interest.

## Publisher’s note

All claims expressed in this article are solely those of the authors and do not necessarily represent those of their affiliated organizations, or those of the publisher, the editors and the reviewers. Any product that may be evaluated in this article, or claim that may be made by its manufacturer, is not guaranteed or endorsed by the publisher.
